# Emotion, proficiency, and arousal: exploring speech and physiological responses in Chinese ESL learners

**DOI:** 10.3389/fnhum.2025.1653894

**Published:** 2025-11-11

**Authors:** Mengjiao Wu, Jennifer M. Roche

**Affiliations:** 1 College of Foreign Languages, Shanghai Maritime University, Shanghai, China; 2 Speech Pathology and Audiology, Kent State University, Kent, OH, United States

**Keywords:** embodied cognition, physiological arousal, L2 speech, electrodermal activity (EDA), acoustic, affective words

## Abstract

**Introduction:**

The coordination and expression of cultural specific affective cues during speech production in a second language (L2) reflects pragmatic adaptation, which is a critical step toward learning and achieving broader pragmatic competence. Embodied cognition provides a framework for understanding how cognitive and emotional processes shape L2 expression.

**Objective:**

This study examined how immersive language experience influences pragmatic adaptation through the vocal expression of affect and physiological arousal in Chinese ESL learners.

**Methods:**

Acoustic analysis and electrodermal activity (EDA) measurements were used to assess affectively valenced word production in speakers with varying levels of immersive English experience.

**Results:**

High-immersion speakers exhibited greater pitch, intensity, and duration variation, enhancing emotional expressivity. Low-immersion speakers showed constrained vocal patterns and significantly higher physiological arousal, likely due to increased cognitive demands and anxiety.

**Discussion:**

These findings highlight the impact of L2 proficiency on affective language embodiment and the cognitive challenges faced by L2 learners. This study offers novel insights by considering a pictorial character-based language, broadening our understanding of emotion-language interaction. Findings have implications for second-language education, cross-cultural communication, and bilingual speech therapy.

## Introduction

Learning a language goes beyond the cognitive processes of acquiring words and grammar, and second language learning can be even more challenging, as mastering socio-pragmatic rules is difficult without an immersive context (e.g., Allami and Naeimi, 2011; Bialystok, 1993; Cook and Liddicoat, 2002). Even native speakers may not be aware of their own pragmatics, as formal education rarely teaches pragmatics as explicitly as vocabulary and grammar (Bardovi-Harlig, 2013; Wolfson, 1989). The ability to coordinate affect and prosody in vocal production provides a unique perspective to pragmatic competence in L2 acquisition. In second language acquisition (SLA), the development of pragmatic competence can be particularly challenging in the absence of formal instruction (Bardovi-Harlig and Dörnyei, 1998; Mokoro, 2024; Wyner, 2014), which makes interpreting the pragmatic function of prosody even harder (Levis, 1999; Celce-Murcia et al., 2010).

Nevertheless, L2 learners may develop pragmatic competence through pragmatic transfer and adaptation (Costa et al., 2008; Kasper, 2001; Trofimovich and Isaacs, 2016), which involves adjusting one’s communicative behavior to fit the social context and using one’s understanding of these cues to guide adaptation in a cross-cultural setting. For instance, adapting one’s affective prosody—a key component of pragmatic competence, encompassing intonation modulation, appropriate pausing and intensity, and timing of affective cues—is a skill L2 learners must develop (Kermad, 2021). They may achieve this by leveraging their understanding of culturally shaped affective cues (Kasper, 2001). Therefore, through pragmatic transfer and adaptation, by aligning their vocal cues with those of cultural counterparts, learners gradually acquire the ability to convey and recognize emotions and attitudes in a non-native cultural context—likely being driven by mechanisms of transfer (Kasper, 2001) and interactive alignment (Costa et al., 2008).

Learners may also draw on inherent cognitive processes, such as emotional processing, which integrates the bi-directional nature of cognitive appraisals with physiological reactions (e.g., *appraisal theory*; Ellsworth, 1991, 2013; Moors et al., 2013; Russell, 2003; Scherer, 1999) through embodiment (Barrett and Lindquist, 2008; Titchener, 1914), such that appraisals and bodily responses interact during emotional processing (Jerath and Beveridge, 2020; Manstead and Wagner, 1981; Nummenmaa et al., 2012; Schachter and Singer, 1962). Leveraging the mind–body interaction during language acquisition, especially in an immersive learning context, L2 (second language) learners may more easily express affectively valenced language (Kosmas and Zaphiris, 2020; Lan et al., 2015). For instance, when learners physically and emotionally engage with language—like feeling joy while using joyful words—it can strengthen the connection between emotion and language, helping them understand and use affective words more quickly through an embodied experience (Kissler and Herbert, 2013; Yu et al., 2021; Scott et al., 2012). While extensive research has demonstrated that immersive contexts promote fluency and competence in L2 speech (e.g., Porter and Castillo, 2023; Freed et al., 2004; Nicolay and Poncelet, 2013; Segalowitz and Freed, 2004), our study contributes to a growing body of literature that supports the notion that immersion and affect interact during lexical access and vocal production and by examining both the subjective (conscious interpretation and spoken representation) and objective experience (non-conscious physiological response) of emotion, supporting Wang et al.’s (2025a) argument that a dynamic, multimodal approach is essential for understanding emotional processes. Adopting an embodied cognition framework, we investigate how physiological responses and prosodic cues influence language production in an L2 context.

## Background

During communicative interactions, pragmatics plays a critical role that shapes language in context, often from a sociocultural framework (Beltrama, 2020; Hasan, 2012; Holmes, 2018). L2 learners may benefit from immersive environments, by learning pragmatic skills from native speakers who facilitate pragmatic rules, thereby strengthening both lexical and contextual use and understanding of the L2 (Bardovi-Harlig and Hartford, 1993; Xiao, 2015). Pragmalinguistic and socio-pragmatic competence is essential for developing culturally and socially appropriate communication skills and enhancing effective cross-cultural exchanges (Byram, 1997; Kinginger and Belz, 2005).

Immersive language environments and real-world interactions not only facilitate the development of pragmatic competence through transfer and adaptation, but also allows language and communication to become embodied—i.e., the mind and body interact to shape our thoughts, actions, and even language (for review, see Barsalou, 2008; Louwerse and Jeuniaux, 2010; Wilson and Golonka, 2013). As language learners learn, they may engage intrinsic cognitive mechanisms (e.g., embodiment, emotional appraisal) that guide the development of strategies to ease language acquisition (Al-Hejin, 2004; Arnold, 2011; Ellis, 2006; MacIntyre and Vincze, 2017). For instance, Louwerse and Jeuniaux (2010) provide evidence for an embodied approach to language processing, demonstrating across four studies that when different facets of a representation—such as semantics and iconicity—are aligned, language processing is facilitated. These findings are consistent with *dual coding theory* (Paivio, 1990), which posits that verbal and non-verbal systems interact to enhance comprehension. Theories of embodied cognition emphasize that cognitive processes are deeply rooted in sensory and motor experiences, suggesting that both concrete and abstract concepts are understood through bodily interactions with the world (Barsalou et al., 2003; Dove, 2014; De Vega et al., 2012). Not only are physical objects understood through our interaction with them, but abstract ideas, like emotions, are grounded in how our body feels and reacts. For instance, abstract words often carry emotional weight, which ties them directly to the body’s responses to the environment (Kousta et al., 2011). This integration of perception, action, and emotion highlights the dynamic collaboration between the brain, body, and environment. Language comprehension and production, therefore, are not isolated mental activities but are intertwined with physical and emotional experiences.

Emotional or affective expression (e.g., facial and vocal gestures; Pell et al., 2009; Sauter et al., 2010; Scherer et al., 2001), which develop before language, play a foundational role in how we understand abstract ideas (Bloom, 1998; Hoemann et al., 2020; Ogren and Johnson, 2021). Through embodied experience, emotions provide a direct, physical connection between words and their meanings, making the process of understanding these concepts more intuitive and grounded in real-world interactions (Kosmas and Zaphiris, 2020; Tillman and Louwerse, 2018). This embodied perspective offers a nuanced understanding of how language becomes deeply connected to human experience. These insights are especially relevant for L2 learners, who must integrate new vocabulary and grammar into their existing embodied frameworks so as to acquire the knowledge of form-function-context mappings (Monaco et al., 2019; Pulvermüller et al., 2005), to foster deeper language integration to achieve pragmatic fluency (Atkinson, 2010; Ayedoun et al., 2019; Graesser et al., 2011).

Focusing on the process of perception and action in real-time can help capture the full impact of embodied processing. While concrete words are easier to grasp., affective elements may actually speed up this process, sometimes making affective representations more easily activated in cognition (Kousta et al., 2011; Kousta et al., 2009). A number of studies have found that emotional words in one’s native language (L1) are often found to evoke faster and stronger responses compared to neutral words, demonstrating a robust emotional word processing advantage (Chen et al., 2015; Conrad et al., 2011; Kousta et al., 2009). This advantage suggests that these words are more deeply embedded in the cognitive and emotional framework of the speaker (Anooshian and Hertel, 1994; Sheikh and Titone, 2016). Furthermore, emotional words in L1 elicit stronger physiological responses, such as increased skin conductance (Harris, 2004; Harris et al., 2006) and EMG (electromyographic) activity (Larsen et al., 2003). These physiological indicators provide support that affectively valenced words are embodied, automatically triggering emotional responses that are integrated into the speaker’s bodily state. As emotions are biologically grounded and are widely recognized across cultures (Ekman, 1992; Pell et al., 2009; Scherer, 2003), there is the potential that L2 learners cognition may be strategically coordinating emotion (i.e., one’s bodily experience) during processing (i.e., recognition), and action (i.e., speaking).

In bilinguals, there are notable differences in how emotional words are processed in L1 compared to L2. Opitz and Degner (2012) found that affective valence of L2 words are processed in a less immediate way, in the context of a highly integrated L1/L2 lexicon. Bilinguals may also *feel* less affected by some pragmatic forms (e.g., swear words) in L2, making it easier to express taboo words, because they do not hold as much cultural significance (e.g., Dewaele, 2004). Similarly, Harris et al. (2003) found that late Turkish-English bilinguals exhibited stronger skin conductance responses (SCRs) to taboo words in L1 compared to L2, highlighting a physiological difference in emotional processing between the two languages. These differences are sometimes attributed to the “disembodied” nature of L2, where emotional experiences are not as deeply integrated due to the typically formal and less emotionally rich contexts in which L2 is learned (Jończyk, 2016; Pavlenko, 2012). This is likely due to the L1 being developed early on with the emotional regulation systems during affective socialization, thus, embedding vocabulary within specific emotional and contextual frameworks may be more tightly coupled in one’s native language (Harris et al., 2006). In contrast, L2 is often acquired in environments that do not foster the same depth of emotional integration, resulting in a larger emotional distance and less embodied emotional responses (Baumeister et al., 2017). This disembodied nature not only affects emotional resonance but may also weaken the pragmatic cues and limits pragmatic transfer and adaptation toward competence in L2 by reducing learners’ ability to interpret and produce culturally and contextually appropriate language in social interactions.

On the other hand, L2 proficiency could also affect how embodied cognition influences emotional processing and pragmatic fluency. Studies indicate that higher proficiency in L2 can lead to a more embodied experience of the language, enhancing emotional responses similar to those in L1 (e.g., Baumeister et al., 2017). However, less fluent L2 speakers are more likely to experience higher cognitive load and anxiety when producing emotional speech in L2 (Chen and Chang, 2004; Liu, 2006; Papi and Khajavy, 2023; Wang et al., 2025a), which could result in increased physiological arousal (Shi et al., 2007). This heightened arousal is linked to the greater effort required to coordinate lexical access and emotional regulation in a less proficient language (Altarriba and Basnight-Brown, 2011; McLaughlin et al., 1983). Furthermore, higher proficiency in L2 can diminish the arousal difference between L1 and L2 by making L2 more embodied. As proficiency increases, L2 learners are better able to integrate affective experiences into their L2 repertoire, reducing the emotional distance and enhancing pragmatic fluency (Harris, 2004).

Thus, developing pragmatic competence in a second language is a complex process that requires integrating linguistic forms, cultural norms, and social appropriateness. Embodied cognition offers a compelling framework to explore how cognitive and emotional processes shape L2 pragmatic learning, processing and fluency, especially for multilingual learners navigating diverse cultural contexts. Additionally, the related literature on embodiment mainly focused on alphabetic L1 and L2s, such as Spanish-English (Sutton et al., 2007; Kazanas and Altarriba, 2016), Turkish-English (Harris et al., 2003), and Greek-English (Eilola and Havelka, 2011). Research attention is needed for bilinguals with logographic L1s, such as Chinese-English bilinguals. Tang et al. (2023) argued for the necessity of evaluating emotionality differences in Chinese and Western languages, because emotions are likely understood and conceptualized differently across languages and cultures. When it comes to Chinese and English, Chinese emotion words are embodied more “interoceptively,” associated with internal bodily sensations, whereas English emotion words are embodied more “autonomically,” linked to automatic physiological responses (Zhou et al., 2022). The differences in how these two languages embody emotion concepts might be related to different attitudes about emotional expressions, with Chinese speakers being more introspective and English speakers more emotionally expressive.

Chinese culture values also tend to be different, as they typically reflect more control and restraint in emotional expression (especially negative emotion), while Western culture tends to appreciate more direct expression of feelings (Butler et al., 2007; Murata et al., 2013; Tsai et al., 2006). Chinese and English speakers may differ significantly in their experience of producing affectively valenced words, raising the question of whether Chinese-English bilinguals have similar emotional experiences across both languages. Despite growing interest in bilingual emotionality, relatively few studies have examined these differences among native Chinese speakers (Chen et al., 2015; Tang et al., 2023). The current study seeks to address this gap and contribute to the limited body of research in this area.

In this study, we explore how the interaction between physiology and cognition interacts to support second language (L2) fluency by examining how native Mandarin speakers’ immersive language experience express emotionally charged words in their native language and non-native L2 English. This study aims to understand how embodied cognition influences language learning and affective expression in L2 speakers. It was hypothesized that (1) speakers would be more aroused when producing affectively valenced words than neutral words in both L1 and L2 because emotional words are more likely to trigger emotion and result in arousal increase. It was also hypothesized that (2) a higher level of L2 immersion would diminish the difference in arousal between L1 and L2, because L2 becomes more embodied as L1 with the increased experience speaking the language in an immersive context, leading to more similar emotional experience and responses.

## Methods

### Participants

A total of 22 participants (mean age = 24.8 yrs., sd = 4.2 yrs.; women = 12; men = 10) were recruited from international students from a Midwestern University in the United States. Of these participants, approximately 12 participants lived in the USA and enrolled in regular university courses for longer than 12 months (mean stay = 4.65 yrs., sd = 2.11 yrs.; high immersive speakers) and 10 participants had been living in the USA for less than 12 months (mean stay = 0.55, sd = 0.28 yrs.; low immersive speakers) and were ESL (English as second language) students at the language center in the same university. According to their self-reports, all participants were born and lived in China until they were 18 yrs. of age. Additionally, 17 participants had complete electrodermal activity (EDA) data, and 16 participants had complete acoustic data—sometimes the devices failed to properly record the sound files and EDA data. All analyses were conducted based on the available complete data for each measure. Participants were compensated with a $5 gift card for every half hour of participation and all speakers had normal-to-normal corrected vision, with no reports or diagnoses of speech or hearing impairments.

### Materials and stimuli

All stimulus presentations and audio recordings were controlled by a Matlab, Psychtoolbox-3 program. All participants were seated in front of a 13-inch Macbook Pro computer and USB CAD U37 Studio Condenser recording microphone. Participants also wore the Empatica E4 sensor, which collected physiological data during the task. Stimuli included bi-syllabic English and Chinese words that shared semantic meaning, which were presented in the middle of the computer screen. The English/Chinese words included 12 negative English/Chinese (e.g., Cancer/癌症—Áizhèng), 12 positive English/Chinese (e.g., Success/成功—Chénggōng), and 24 neutral English/Chinese (e.g., Pencil/铅笔—Qiānbǐ) affectively valenced words chosen from the Affective Norms for English words (ANEW; Bradley and Lang, 1999). The bi-syllabic English words were chosen from the ANEW database, based on valence ratings; positive (mean = 8.29), negative (mean = 1.78), neutral (mean = 5.17). Once these words were chosen, the English words were translated into the Chinese corollary and characters by a native speaker of Chinese. The Chinese translation of each word was also limited to two syllables. The experimental task also included two short authentic passages of around 150 words that were presented at the start of each experimental block: one passage in Chinese on how to cook rice, and the other in English on how to select teaching materials for reading.

### Design and procedure

In this task, participants were presented with a total of 100 affectively valenced Chinese and English words (4 practice trials; 96 experimental trials) in the middle of a computer screen, one word at a time. The word would disappear after being presented for 3 s. Participants would then be instructed to speak the word twice into the microphone after hearing a beep. They were also instructed to press the “spacebar” on the keyboard to end the recording and advance to the next word.

Prior to the start of the task, the experimenter placed the Empatica E4 sensor on the participant’s left wrist (all participants were right-handed). For approximately 10 min prior to the experimental task, the participant completed a task on the computer unrelated to the current task. This allowed us to acquire a more accurate reading of the participant’s physiological state, as the participant was able to get comfortable and remained in a fixed position prior to the beginning of this experimental task. This was an important methodological consideration, as moving too much and any anxiety from wearing unfamiliar equipment can impact the measures collected from the E4 sensor. The participant was asked to keep their left hand stable and flat on the computer table, but were allowed to move their right hand to manipulate the computer keyboard to transition through the experimental trials (i.e., ‘spacebar’ keypress when finished recording). To begin an experimental block (language x affective valence), speakers were first presented with a short non-affectively valenced passage in the language condition they were currently in (e.g., a passage in Chinese or a passage in English). This was done as a means to *activate* the L1 or L2 language system, as to control for any physiological or cognitive costs incurred from switching between the language categories. A practice trial consisting of 4 practice words was presented for practice before the main task started.

Participants were randomly assigned to one of four between-subjects conditions: 2 Language Order (English/Chinese or Chinese/English) x 2 Affect Condition Order (Positive first v. Negative first). The Language Order between subjects condition indicates which language condition came first: English/Chinese—English words came first; Chinese/English—Chinese words came first. Additionally, Affect was counterbalanced between subjects, in that participants were randomly assigned to produce the Positive words first, and others were required to produce the Negative words first. This resulted in eight experimental blocks of trials. For example, if a participant was assigned to the Chinese and positive first condition, their trial structure included (1) 12 positive valenced words to be spoken in Chinese, with positive prosody (i.e., tone of voice), (2) 12 neutral valence words to be spoken in Chinese, with neutral prosody, (3) 12 positive valenced words to be spoken in English, with positive prosody (4) 12 neutral words to be spoken in English, with a neutral prosody. This was then repeated with negative prosody in Chinese first and then English. A similar structure was implemented for the other three between subjects Language x Affective Prosody conditions, in which language and affect were counterbalanced between participants.

### Measures

#### Acoustic variation

The most commonly evaluated acoustic correlates of affective expressions include measures of timing, intensity, and pitch (Juslin and Laukka, 2003; Scherer, 2003). As an estimate of speaker expressiveness, we recorded and composited measures of duration (timing; msec), intensity (amplitude; dB), and pitch (*fo*; Hz). Each of these measures were collected using the standard aggregating features in Praat (Boersma and Weenink, 2005). It should be considered that when measuring speech, researchers should consider the type of aggregation method, especially related to pitch. For instance, Strik and Boves (1991) provide a compelling argument to use non-linear aggregation of pitch over time as a means to reduce signal variability caused by differing speaking rates. While this is a common technique, we chose to use linear aggregating methods, because we were explicitly interested in affect and the relation between speaking rate and pitch should be preserved in the signal—because they are both important cues. Using non-linear averaging for pitch across the time series while retaining duration as a predictor allows us to capture interactions between pitch and temporal dynamics that may carry emotional information. Non-linear aggregation might obscure these effects, potentially masking relevant affective signals. Our focus was not on the specific communicative content of these acoustic variations but rather on the differences in variation across conditions, therefore, a composite was used to evaluate these cues as they are highly correlated and address general variation in the affective expressions.

#### Electrodermal activity (EDA)

EDA was collected using the Empatica E4 wristband sensor from participants during the course of the experiment. The Empatica E4 sensor is sampled at 4 Hz for EDA, which means it collects EDA data four times per second. EDA, a measure of physiological arousal has been frequently used as a correlate of emotional arousal and cognitive load. EDA is preferred to other measures of arousal because it has been suggested to be very sensitive and under strict control of the sympathetic (involuntary) nervous system (e.g., Cacioppo et al., 2007; Sequeira et al., 2009), therefore being recognized as one of the most sensitive physiological measures of emotional and cognitive activation (Eilola and Havelka, 2011; Hugdahl, 1995). During data collection, a Matlab Psychtoolbox-3 program controlled stimulus presentation, printed a time-stamp of when the stimulus (affectively valenced word) appeared on the computer screen and when the participant pressed the spacebar on the computer’s keyboard. This allowed us to time match the participant’s trial level data with the EDA data.

### Analytic approach

Linear mixed random effects models were used due to their ability to account for both fixed and random effects, because this approach provides more flexibility compared to traditional ANOVA, as it can accommodate variability at multiple levels (e.g., at the subject and item level; Baayen et al., 2008; Barr et al., 2013). To do this, a fully maximal random effects model was always attempted with both fixed effects (predictors of interest) and random effects (random intercepts or slopes for subjects and items). We then employed a backwards removal of random effects until model convergence was met. We then compared the model that converged against the intercept only model, to ensure the model selected produced the best fit to the data.

## Results

### Acoustic variation

**Figure 1:**
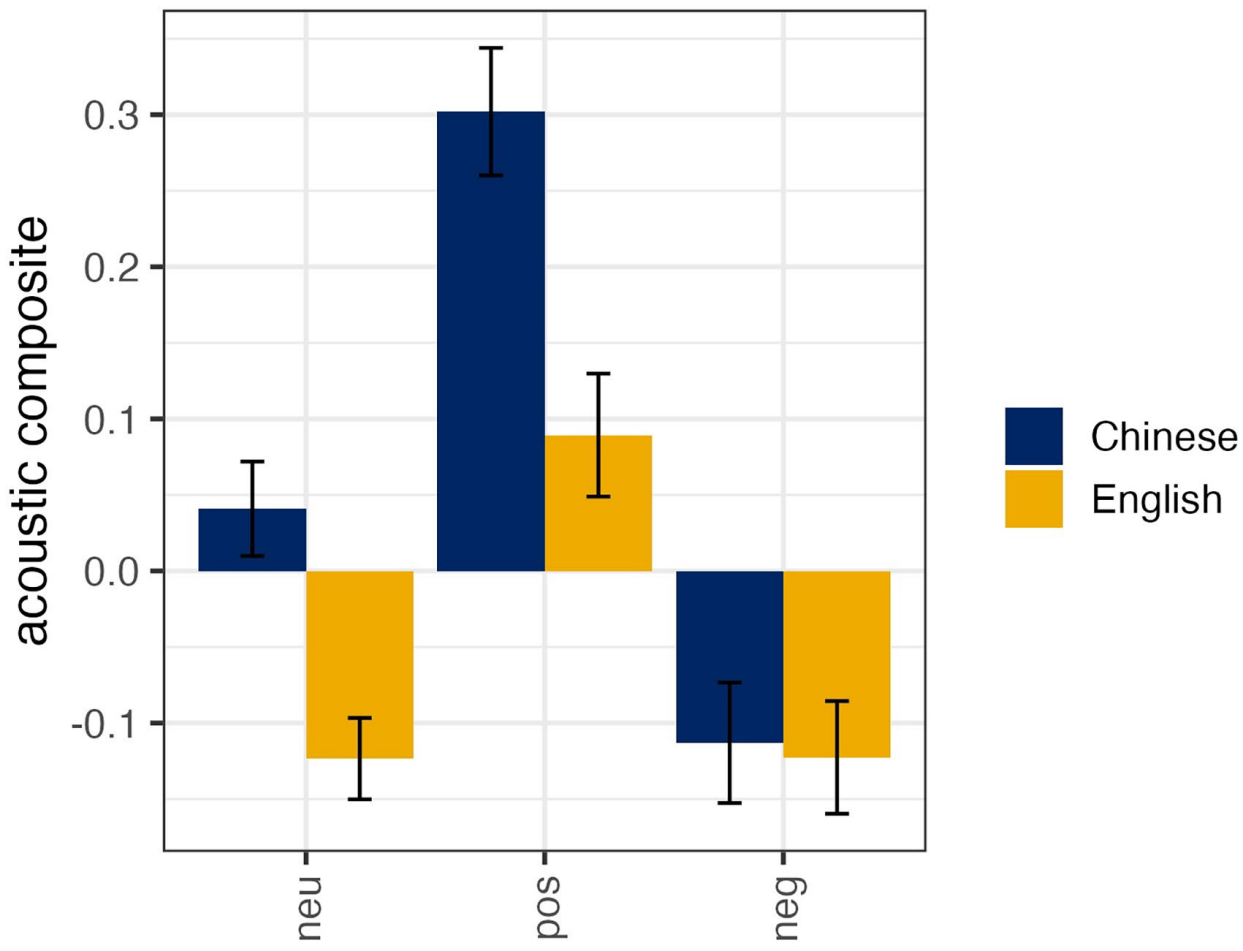
Mean acoustic composite scores (duration, intensity, and pitch [fo]) with standard errors for the two-way interaction effect associated with the Language (Chinese, English) by Affective Expressions (neutral, negative, positive). Higher composite values indicate longer durations, greater vocal intensity, and higher pitch—this is most clearly seen in positive Chinese utterances.

A linear mixed random effects model was used to evaluate the composite of acoustic variation (duration, vocal intensity, and *fo*) as a function of language spoken, affective expression, and immersion. Subject and item were set as random intercepts and language spoken and affective expression were modeled as the random slopes on the subject intercept—a fully maximal random effect structure did not permit model convergence. However, this model did produce a significantly better fit than an intercept only model—*x*^2^ = 69.12, *p* < 0.001, *AIC* = 1063.5. The chosen model accounted for approximately 73.4% (*R*^2^) of the variance in the acoustic composite score. Results indicated a main effect of language spoken, affect type, immersive experience, and an interaction between language spoken and affective expression. In light of the higher order interaction, only the interaction and main effects not involved in the interaction are reported. The main effect of immersive experience (*ß* = −0.51, *SE* = 0.23, *t* = −2.20, *p* = 0.03) indicated that participants with longer immersive experiences (i.e., longer than 12 months) had more positive composite scores (i.e., longer durations, higher intensities and pitch) than the speakers with shorter immersive experiences (i.e., less than 12 months). Additionally, speakers varied their acoustics associated with neutral (*ß* = 0.15, *SE* = 0.05, *t* = 3.28, *p* < 0.01) and positive utterances (*ß* = 0.21, *SE* = 0.07, *t* = 3.19, *p* < 0.01), but not negative utterances (*ß* = 0.02, *SE* = 0.06, *t* = 0.24, *p* = 0.81; see Figure 1). This suggests that how people speak—specifically their pitch, intensity, and timing—seems to depend on the language they are using, the emotion they are expressing, and the extent of their immersive experience.

### Electrodermal activity (EDA)

A linear mixed random effects model was used to evaluate the electrodermal activity (EDA) as a function of language spoken, affective expression, and length immersion experience. Subject was set as random intercepts and language spoken and affective expression were modeled as the random slopes on the subject intercept. Item was dropped from the model and the fully maximal random effect structure did not permit model convergence. However, the selected model did produce a significantly better fit than an intercept only model—*x*^2^ = 425.68, *p* < 0.001, *AIC* = 1176.6, and accounted for approximately 91% (*R*^2^) of the variance in the electrodermal activity. Results indicated a 3-way interaction between language spoken, affective expression, and length of experience.

**Figure 2:**
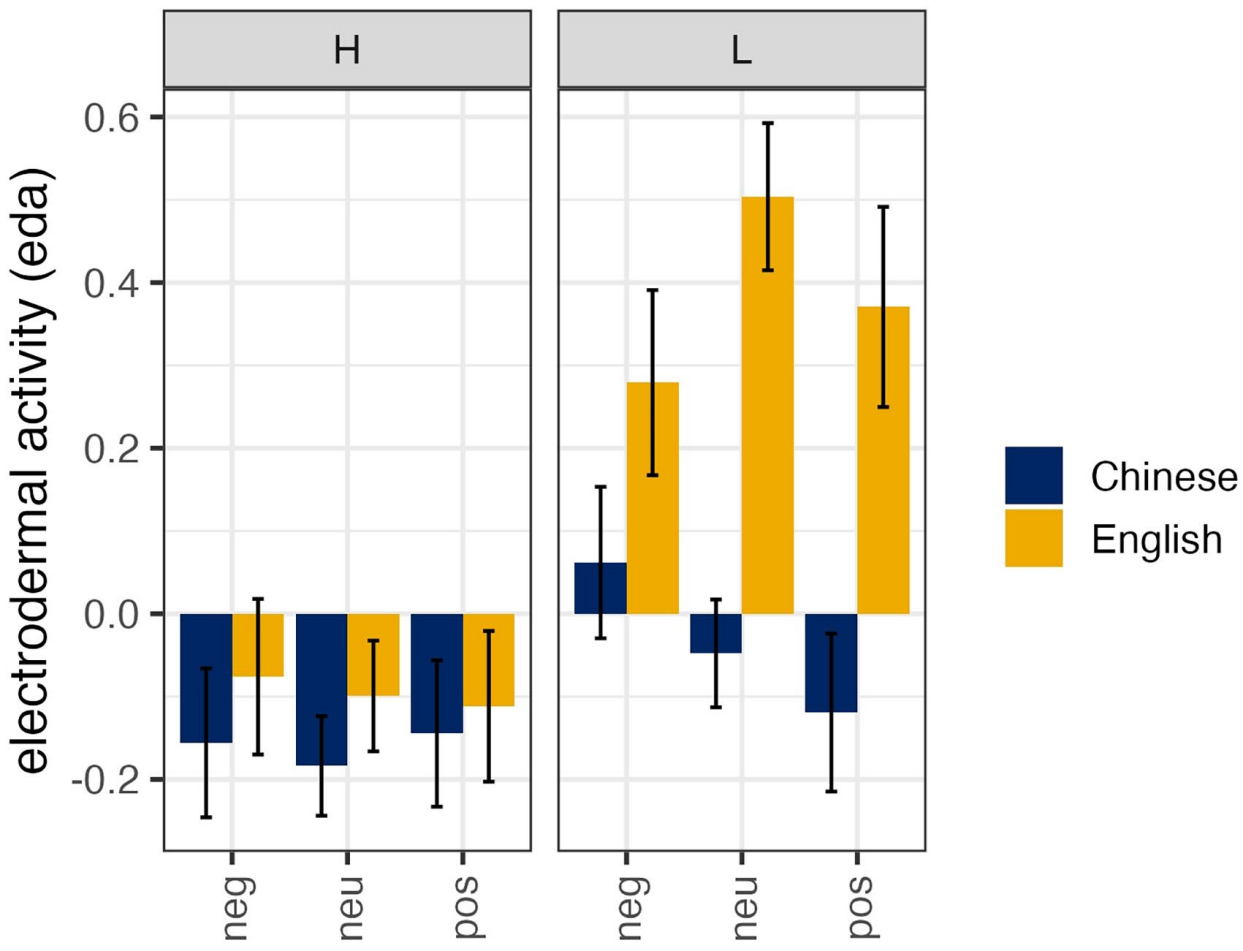
Mean electrodermal activity (EDA) with standard errors across languages (Chinese, English), affective expressions (neutral, negative, positive), and immersive experience length (high >12 months, low <12 months). Positive values indicate higher physiological arousal. The figure has two panels for immersion level (high = H, low = L), showing most prominently that low-immersion participants exhibited the highest EDA for English neutral words.

As seen in Figure 2, participants with less immersive experience experienced significantly higher EDA when producing neutral (*ß* = 0.55, *SE* = 0.12, *t* = 4.25, *p* < 0.001) and positive words (*ß* = 0.50, *SE* = 0.13, *t* = 3.70, *p* < 0.01) in English, relative to Chinese. Additionally, the participants with less immersive experience experienced higher EDA when producing negative words (relative to neutral) in Chinese (*ß* = 0.13, *SE* = 0.06, *t* = 2.26, *p* = 0.03), but higher EDA when producing neutral words in English relative to negative words (*ß* = −0.22, *SE* = 0.06, *t* = −3.84, *p* < 0.001; see Figure 2). This suggests that the speakers in this sample with less experience in an immersive language environment showed different physiological responses depending on the language they were speaking and the type of emotional expression they were producing.

Participants with high immersive experience showed no significant difference in EDA between Chinese and English across all affective conditions (negative/neutral/positive). Notably, affective valence (neutral vs. negative) did not modulate EDA in either language among participants with high immersive experience. This suggests that prolonged immersion may reduce physiological arousal differences between the languages they were speaking and among the types of emotional expression they were producing, resulting in uniform EDA responses regardless of linguistic or emotional context.

## Discussion

The findings from the current study provided support to the notion that an immersive L2 speaking experience importantly extends beyond the words learned (Bardovi-Harlig and Hartford, 1993; Kosmas and Zaphiris, 2020; Lan et al., 2015; Porter and Castillo, 2023; Xiao, 2015), as the immersive experience may be critical to shape how emotions are represented, expressed, and experienced (e.g., important aspects of pragmatic competence—Rafieyan and Rozycki, 2019; Kissler and Herbert, 2013; Yu et al., 2021; Scott et al., 2012). The results of the study showed that length of immersion differentially influenced both outward (vocal affect) and inward (physiological arousal) experiences of emotion during language production (consistent with Barsalou, 2008; Louwerse and Jeuniaux, 2010; Wilson and Golonka, 2013). The immersive L2 context elicited affective utterances resembling the more vocally expressive style of American speakers (Ip et al., 2021), potentially reflecting mechanisms of transfer and adaptation that support the development of pragmatic competence.

In fact, speakers having been immersed in an L2 context for a longer period of time tended to be more vocally expressive: more acoustic variation in pitch, intensity, and duration. However, these participants exhibited a more attenuated physiological response, as they exhibited comparable EDA levels between English and Chinese across all affective conditions (negative/neutral/positive). This might indicate that sustained exposure to an L2 language environment may reduce the physiological burden of language switching (Altarriba and Basnight-Brown, 2011; Chen and Chang, 2004; Liu, 2006; Papi and Khajavy, 2023). L2 speakers with high immersive experience may have developed automated emotional and linguistic integration, minimizing cross-language physiological responses (consistent with Harris, 2004; Shi et al., 2007).

The opposite occurred for individuals with shorter immersion experiences, such that their vocal expressions were more constrained acoustically (consistent with Dewaele, 2004; Thoma and Baum, 2019). However, the low immersion participants had a much more pronounced physiological response than the high immersion participants. The low immersion group tended to exhibit heightened arousal when speaking in their non-dominant language. This was particularly evident when they produced neutral and positive utterances in English. When speaking Mandarin Chinese, however, negative utterances elicited the strongest arousal response. This might suggest that cross-cultural differences in emotion suppression norms may be evident, given that Chinese culture may impose stronger social constraints on negative emotional expression than English-speaking Western cultures (Murata et al., 2013; Tsai et al., 2006). While the literature suggests that negative stimuli elicit heightened arousal across cultures (suggesting arousal to negative stimuli to be universal; Ho et al., 2015; Järvelä et al., 2021; Naranowicz et al., 2022), the degree of reactivity may be shaped by cultural norms. For instance, US Americans often report higher emotional reactivity to negative visual stimuli than their Chinese counterparts, and in China, cultural norms encourage suppression of overt expressions of negative emotion (Liddell and Williams, 2019; Huwaë and Schaafsma, 2018; Tyra et al., 2024). Notably, research shows that when overt suppression is required, physiological arousal can increase (Gross and Levenson, 1993; Peters et al., 2014). This supports the idea that, across cultures, negative affect may universally trigger heightened physiological arousal (Hermanto et al., 2012; Zhang et al., 2021), even if outward expression is muted. In immersive contexts, however, cultural practices may be carried into the setting (Kim, 2017), allowing for more overt expression of negative affect, which could facilitate a release of arousal when experiencing negatively valenced stimuli (Gross and Levenson, 1993; Kennedy-Moore and Watson, 2001; Thakur et al., 2017).

Individuals in the low-immersion group may have experienced heightened arousal when violating these norms. Emotional engagement in speech seems to be shaped not only by one’s ability to produce the words of a language, but also by the cultural and linguistic norms that may influence how speakers regulate emotion across languages. Language processing and affective expression are dynamically influenced by the duration of immersion, highlighting the interplay between bodily states and language production (Kosmas and Zaphiris, 2020; Tillman and Louwerse, 2018).

Assessing variation in both acoustics and EDA offers valuable insights into how the cognitive system represents and supports L2 language learning and pragmatic competence, leading to a better understanding of cross-cultural communication. In early stages of L2 language learning, non-native speakers may experience greater emotional and cognitive effort when expressing affect (Chen and Chang, 2004; Liu, 2006; Papi and Khajavy, 2023), − as both lexical access is generally more difficult and they may experience stress responses associated with public speaking, interpersonal interactions, and even clinical contexts such as speech therapy (Roseberry-McKibbin et al., 2005). While the current study cannot explicitly address the mechanism that elicits greater cognitive load, we do see that the Low immersion group is under greater cognitive strain. Understanding how language and pragmatic competence shape affective expression and physiological response could inform educational approaches for language learning, as well as improving speech recognition models that aim to capture pragmatic nuance in bilingual speakers.

From an embodied cognition view, these findings support the notion that cognitive processes are deeply rooted in the body’s sensory and motor systems (Barsalou, 2008; Barsalou et al., 2003; Dove, 2014; Winkielman et al., 2015). The observed acoustic variation highlights how speech production is not just a cognitive act but one that engages sensorimotor mechanisms (Baumeister et al., 2017; Foroni, 2015; Foroni and Semin, 2009; Kousta et al., 2011; Larsen et al., 2003), reinforcing the idea that emotion is not simply encoded abstractly in the brain but is enacted through the body (Baumeister et al., 2017; Dimberg et al., 2000). Similarly, the physiological responses reveal that language processing is intertwined with bodily arousal, suggesting that emotion is not merely understood but physically felt (Chen et al., 2015; Conrad et al., 2011; Harris, 2004; Harris et al., 2006; Kousta et al., 2009).

Ultimately, this research underscores the complex connection between language, pragmatics, cognition, and the body (Baumeister et al., 2017; Louwerse and Jeuniaux, 2010; Wilson and Golonka, 2013). Affective expression in speech is not just a matter of vocal output but reflects a dynamic interaction between linguistic and pragmatic experience, as well as motor control, and physiological states (Barsalou et al., 2003; Dove, 2014; Harris et al., 2006; Kousta et al., 2011; Porter and Castillo, 2023). This deeper understanding of how language and pragmatic competence shapes both vocal expression and bodily responses contributes to broader theories of bilingualism, emotion, and the embodied nature of communication, with meaningful implications for education, technology, and clinical practice.

### Limitations and future directions

Like many studies in speech production research, this study had a relatively small sample size, because speech production effects are typically robust within participant; however, it remains consistent with other production studies in the field (e.g., see Ferguson and Kewley-Port, 2007), where detailed acoustic and physiological measures require intensive data collection and analysis. In the current study, because we are also using EDA as a measure, we increased the sample size and based on a power analysis, we were sufficiently powered—with well over 1,500 data points in our sample and the power analysis calling for a minimum of 12 participants needed for statistical sensitivity. We should note, however, that some data loss occurred due to device failure, a common challenge in studies collecting physiological measures such as electrodermal activity (Boucsein, 2012, p. 245; Braithwaite et al., 2013), but given the sample size and repeated measures component, we believe the findings remain meaningful and interpretable.

We should also draw attention to the fact that our design permitted participants to control their response window (on average 3.9 s (low immersion group); 4.6 (high immersion group). EDA has a relatively slow rise time, with peak responses typically occurring around 6 s post-stimulus. This means our measured EDA response may under-estimate the true peak amplitude, potentially introducing measurement error and the absolute magnitudes should be interpreted with caution. However, it is still notable that the Low Immersion group exhibited higher EDA despite having shorter trial durations. If measurement truncation biased our data, it would be expected to attenuate rather than inflate group differences. Thus, the direction and robustness of our findings are unlikely to be explained solely by timing limitations.

While the results provide valuable insight into the interaction between language experience, emotion, and physiological response, it is unclear whether these findings would generalize to other languages or if they are specific to the linguistic and cultural background of the sample. Nevertheless, this study provides a framework that evaluates the dynamic interplay between subjective and objective experience of emotion (see Wang et al., 2025b). In addition, we chose a linear aggregation method instead of the sometimes recommended non-linear methods (see Strik and Boves, 1991). This was a strategic move, but aggregating has the potential of leading to oversimplification, especially when trying to understand the dynamics of emotions. In the current study, we deliberately employed aggregation methods to maintain the acoustic integrity of speech and ensure reliable measurement of affect and physiological aspects of embodied emotion. Unlike studies that track emotions across extended real-world contexts—which can introduce additional variability and noise—we focused on short bursts of subjective and objective emotional responses in carefully controlled recording sessions, preserving the natural data stream while balancing ecological validity and experimental rigor. It should be noted, however, that when adopting a multimodal approach, researchers must make careful methodological choices to preserve the richness of the data and avoid oversimplification, as Wang et al. (2025b) emphasize. This is important because oversimplifying can obscure meaningful patterns in the interactions between modalities, potentially leading to inaccurate or incomplete interpretations. To that end, these limitations are typical of experimental research in this area and do not diminish the overall contributions of the study, but rather highlight areas for future investigation.

## Conclusion

Understanding how L2 learners represent and express affective language can help clinicians tailor therapy approaches for bilingual individuals. Speech intelligibility and prosody play crucial roles in conveying meaning and emotion, and understanding that shorter immersive experiences may lead to weaker language and pragmatic skills is a useful perspective taking tool for clinicians to use, to help them avoid misinterpretation and implement strategies that reduce anxiety and enhance confidence in L2 learners. Ultimately, this research bridges cognitive science, bilingualism, and clinical practice, offering valuable perspectives on the importance of immersive experiences on communication.

## Data Availability

The raw data supporting the conclusions of this article will be made available by the authors, without undue reservation.
